# Preoperative identification of the risk factors of cervical lymph node metastasis in medullary thyroid carcinoma

**DOI:** 10.3389/fendo.2025.1576955

**Published:** 2025-08-21

**Authors:** Yitong Wang, Jiahui Chen, Xuemeng Gao, Ying Huang

**Affiliations:** Department of Ultrasound, Shengjing Hospital, China Medical University, Shenyang, Liaoning, China

**Keywords:** medullary thyroid carcinoma, metastasis, radiomics, multivariate regression model, ultrasound

## Abstract

This research aimed to investigate the preoperative risk factors for lymph node metastasis (LNM) in medullary thyroid carcinoma (MTC) using clinical, pathological, serological, ultrasound, and radiomics characteristics. Additionally, it aimed to explore the diagnostic precision of ultrasound (US) for MTC and LNM. A retrospective analysis of 111 nodules was eligible from 104 patients from January 1, 2000, to December 28, 2024. Based on the presence of LNM, they were divided into Group 1 (with LNM, n=51 nodules from 44 patients) and Group 2 (without LNM, n=60 nodules from 60 patients). Three predictive models were constructed: (1) Model 1: incorporating clinical, pathological, serological, ultrasound features; (2) Model 2: utilizing only radiomics features; and (3) Model 3: combining all the above features. The AUC values for the three models were 0.759, 0.97, and 0.97, respectively. The nomogram for Model 1 achieved a C-index of 0.707. Additionally, we evaluated the diagnostic efficacy of ultrasound for MTC, lymph node enlargement, and metastasis. Results indicated that patients with symptoms on admission, multifocality, and solid lesions in MTC were at increased risk of LNM. The nomogram and radiomics features significantly improved the predictive performance. Our study provides a strong basis for predicting LNM.

## 1 Introduction

Medullary thyroid cancer (MTC) originates from parafollicular C‐cells of the thyroid (1). Due to the neuroendocrine features of C‐cells, patients with MTC may have symptoms such as palpitations, flushing, and diarrhea (2). Hoarseness and dyspnea may occur when the malignancy progresses beyond the thyroid and infiltrates other cervical structures. Although it is an uncommon malignancy, its mortality has been reported as high as 15% (3). Lymph node metastasis (LNM) is closely associated with reduced survival rates and increased recurrence rates in patients with MTC (4, 5).

The main contention focuses on the need for prophylactic lateral neck dissection of patients. The performance of radical neck lymph node dissection increases the incidence of postoperative complications (6), whereas incomplete lymphadenectomy elevates the risks of recurrence and reoperation (7). Therefore, accurate preoperative detection of lymph node metastasis is pivotal for minimizing undertreatment or overtreatment in MTC patients. Additionally, the burden of LNM holds significant prognostic value in predicting survival following surgery and is incorporated into the pathologic staging of disease (8–10). Therefore, it is essential to identify LNM of MTC before surgery.

Currently, the determination of LNM primarily relies on histological analysis. Although this method is effective, it carries risks of hemorrhage and nerve injury. There is an urgent need for a non-invasive method to accurately predict LNM before surgery, as there have already been relevant studies on the predictive factors for LNM in MTC. Some possible factors include gender (11), tumor size, multifocality (12), extrathyroidal extension (13), preoperative calcitonin (Ctn) level, and carcinoembryonic antigen (CEA) level. However, symptoms were rarely included in the current models. Radiomics, a promising technology for exploring LNM, has been focused on some studies (14, 15). But there are a few comprehensive models that combine serological, clinical and radiomics features in the current research. Therefore, the purpose of this retrospective, single-centric study is to investigate the risk factors of LNM in terms of clinical, pathological, serological, ultrasound and radiomics features in MTC, and to explore the accuracy of US in the diagnosis of MTC and LNM.

## 2 Materials and methods

This study was approved by the institutional ethics committee of Shengjing Hospital of China Medical University. Informed patient consent was waived for this retrospective study. (Approval number: 2025PS088K).

### 2.1 Patients and study design

This retrospective study was conducted on a consecutive cohort of patients pathologically diagnosed with MTC from Shengjing Hospital of China Medical University from January 1, 2000 to December 28, 2024. Inclusion criteria were as follows: (1) Patients received thyroid surgery for the first time in our hospital. (2) Patients had clear preoperative ultrasound images and reports. (3) Patients were confirmed as MTC by fine-needle aspiration or thyroidectomy. All patients either underwent thyroidectomy directly or subsequently underwent thyroidectomy after fine-needle aspiration biopsy to confirm the diagnosis of MTC. Exclusion criteria were as follows: (1) Patients with a previous history of thyroidectomy. (2) Patients with absent ultrasound images. If there were more than one MTC nodule in a patient, nodules on different sides proven to be MTC were included separately.

**Figure 1:**
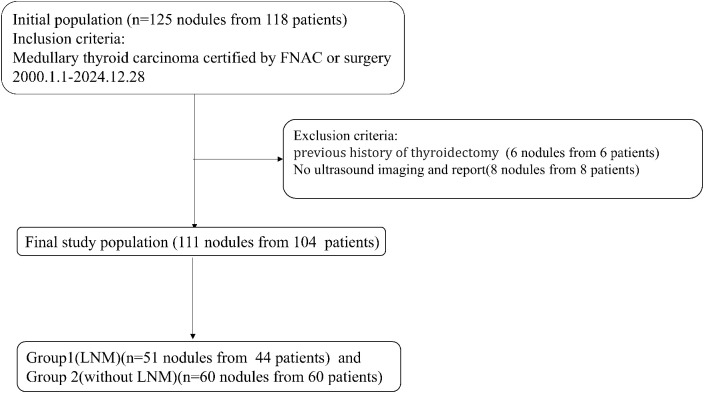
The flowchart of the inclusion and exclusion of patients.

These nodules were divided into Group 1 and Group 2 according to the presence of LNM (**Figure 1**).

### 2.2 Ultrasound measurement

A Philips IU Elite and an IU 22 with a 7 to 9MHz linear-array probe (Beijing, China), a Hivision 900 with a 6 to 14MHz linear-array probe (Tokyo, Japan), TOSHIBA Aplio400 and an Aplio XG SSA-790A with a 7 to 10MHz linear-array probe (Tokyo, Japan), and an ESAOTE MY Lab Twice ultrasonic instrument with a linear array probe of 6 to 15 MHz (California, USA) were used.

Ultrasound features including size of thyroid on the side of the lesion (maximum diameter), size of lesions (maximum diameter), orientation (taller-than-wide or wider-than-tall), composition (solid or not), echotexture (heterogeneous or homogeneous), margin (circumscribed or not), echogenicity (hypoechoic or not), vascularity (abundant or not), with or without echogenic foci (EF), the size of echogenic foci (EFS), and with or without macrocalcifications were collected. Macrocalcifications are defined as a hyperechoic area larger than 1 mm.

Lymph node enlargement is defined as the short-axis diameter >10 mm (16, 17). Based on ultrasound detection of swollen lymph nodes, cohort 1 was divided into positive 1 and negative 1. According to whether the ultrasound doctor could clearly report malignancy in the report, it was divided into positive 2 and negative 2.

Ultrasonic diagnostic indicators included sensitivity, specificity, accuracy, positive predictive value (PPV), and negative predictive value (NPV), positive likelihood ratio (PLR), and negative likelihood ratio (NLR).

### 2.3 Other features for collection and model constructions

Clinical features including age, gender, symptoms on admission, duration of symptoms, and duration of lesion detection were collected. The absence of symptoms on admission was defined as the incidental discovery of the existence of thyroid nodules.

Pathological features including thyroid gland elasticity (hard or not), and extrathyroidal extension (ETE or not) were collected.

Serological features including Free Triiodo-thyroninen (FT3), Free Thyroxine (FT4), thyroid-stimulating Hormone (TSH), Anti-Thyroglobulin Antibody (Anti-Tg), Anti-Thyroid Peroxidase Antibody (Anti-TPO), CEA, and Ctn were collected.

Considering the clinical significance of CEA and Ctn, we used Little’s MCAR test to examine the missing data patterns, e.g., Missing Completely at Random (MCAR) and Missing Not at Random (MNAR). We performed imputation according to its patterns and conducted a univariate analysis to observe the statistical significance after imputation.

**Figure 2:**
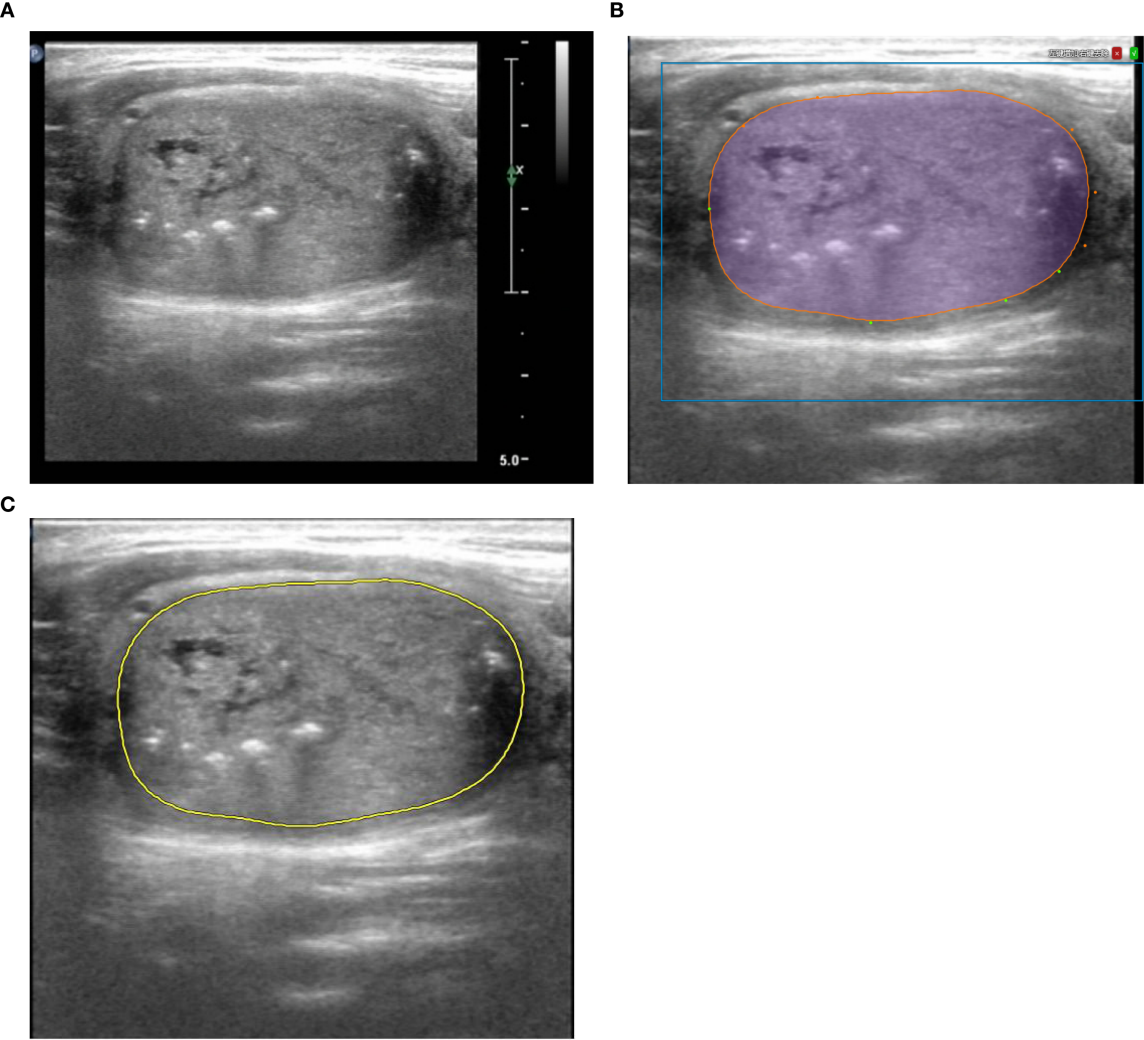
An example of contouring ROI in a 42 - year - old male patient diagnosed with MTC*. **(A)** The cross-sectional image with the maximum diameter. **(B)** Artificial Intelligence on the Darwin platform assisted in contouring, and a physician modified the outline. And **(C)** a senior physician confirmed it. *ROI, Region of Interest; MTC, Medullary thyroid carcinoma.

The cross-sectional image with the maximum diameter of the lesion was selected to contour ROIs. After the AI on the Darwin platform assisted in contouring, a physician with three years of ultrasound diagnostic experience modified the outline. Subsequently, a senior physician with over 20 years of experience reviewed and confirmed it. The process was repeated following a one-week washout period. A third physician would be consulted for discussion and consensus in case of discrepancies. The lesion contouring of a 42-year-old male is shown in **Figure 2**.

Radiomics features were extracted and machine learning algorithms were calculated by Darwin (offline version, developed by marketing@yizhun-ai.com). Machine learning algorithms comprised K - Nearest Neighbours (KNN), Support Vector Machine (SVM), Logistic Regression, Decision Tree, Gradient Boosting Tree (GBT), Random Forest, and Extreme Gradient Boosting (XGBOOST).

**Figure 3:**
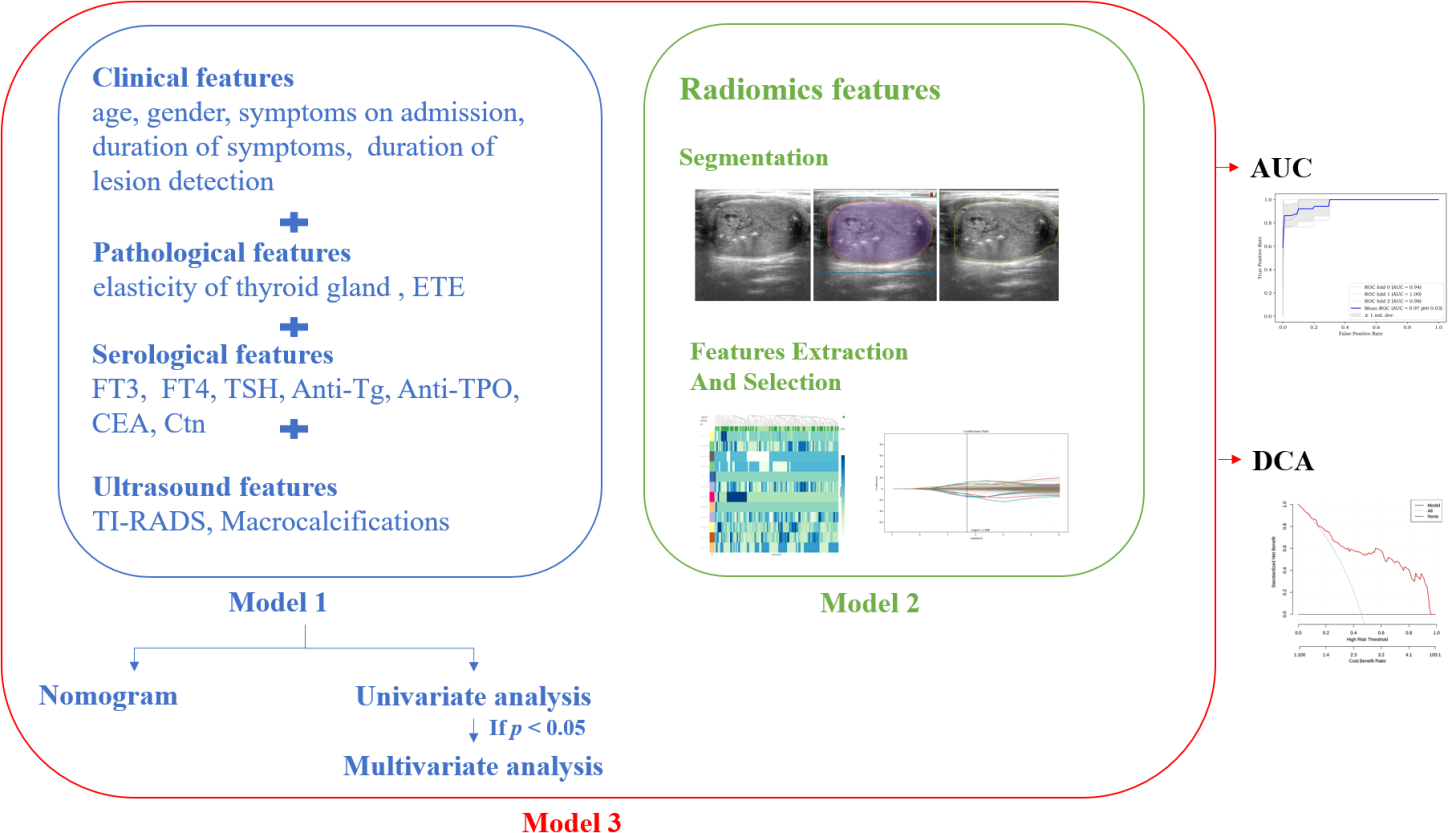
The technical flowchart of the methodology.

Three models were constructed in our study: (1) Model 1: a model with clinical, pathological, serological, ultrasound features; (2) Model 2: a model with only radiomics features; and (3) Model 3: a model mixed with all the above features. After building the models, K-fold cross-validation was employed to test them, with k being set at 3. **Figure 3** shows the technical flowchart of the methodology.

Nomograms were made by using R (version 4.3.3, http://www.r-project.org/).

### 2.4 Statistical analysis

Statistical analysis was performed using IBM SPSS Statistics 25 software (https://www.ibm.com/cn-zh/products/spss-statistics). Continuous data were presented as means ± standard deviations (SD) or medians (M) and interquartile range (IQR). If the *p*-value of a variable in univariate analysis was less than 0.05, it was included in the multivariate logistic regression. Multivariate logistic regression analyses were performed with the aim of identifying independent risk factors for LNM. The Hosmer-Lemeshow test was used to test the model. The Delong test was used to compare different AUC. A *p*-value less than 0.05 is considered statistically significant.

## 3 Results

### 3.1 Baseline Characteristics of Group 1 and Group 2

A total of 111 nodules were eligible from 104 patients with MTC. The age at diagnosis was 53.09 ± 12.29 years, and 66 patients (63.46%) were female. Baseline characteristics of Group 1 and Group 2 are shown in **Table 1**.

**Table 1:** Baseline characteristics of Group 1 and Group 2.

Variable	Total (n=104 patients)	Group1 (LNM) (n=44 patients)	Group2 (without LNM) (n=60 patients)	*P-*value
Age at diagnosis (mean ± SD)	53.09 ± 12.29	50.84 ± 14.08	54.73 ± 10.61	0.114
Gender (n, %)	38M (36.54) 66F (63.46)	20M (45.45) 24F (54.55)	18M (30.0) 42F (70.0)	0.108
Tumor size				0.463
≤2 cm	48	22	26	
2.1–4 cm	50	25	25	
>4 cm	13	4	9	

LNM, lymph node metastasis; F, female; M, male; SD, standard deviations.

### 3.2 Composition of symptoms

Among the 104 patients, a total of 23 patients (22.12%) presented with symptoms, including 17 (73.91%) patients in Group 1 and 6 (26.09%) patients in Group 2. Among the 23 patients, 3 (13.04%) experienced neck discomfort or pain accompanied by tenderness. 1 (4.35%) experienced tenderness and fever. 11 (47.83%) had only neck discomfort or pain. 3 (13.04%) had only tenderness. 2 (8.70%) had only bilateral eyelid edema. Additionally, there was one case (4.35%) each of fever, flushing, and an increase in stool frequency.

### 3.3 Independent risk factors for LNM

**Figure 4:**
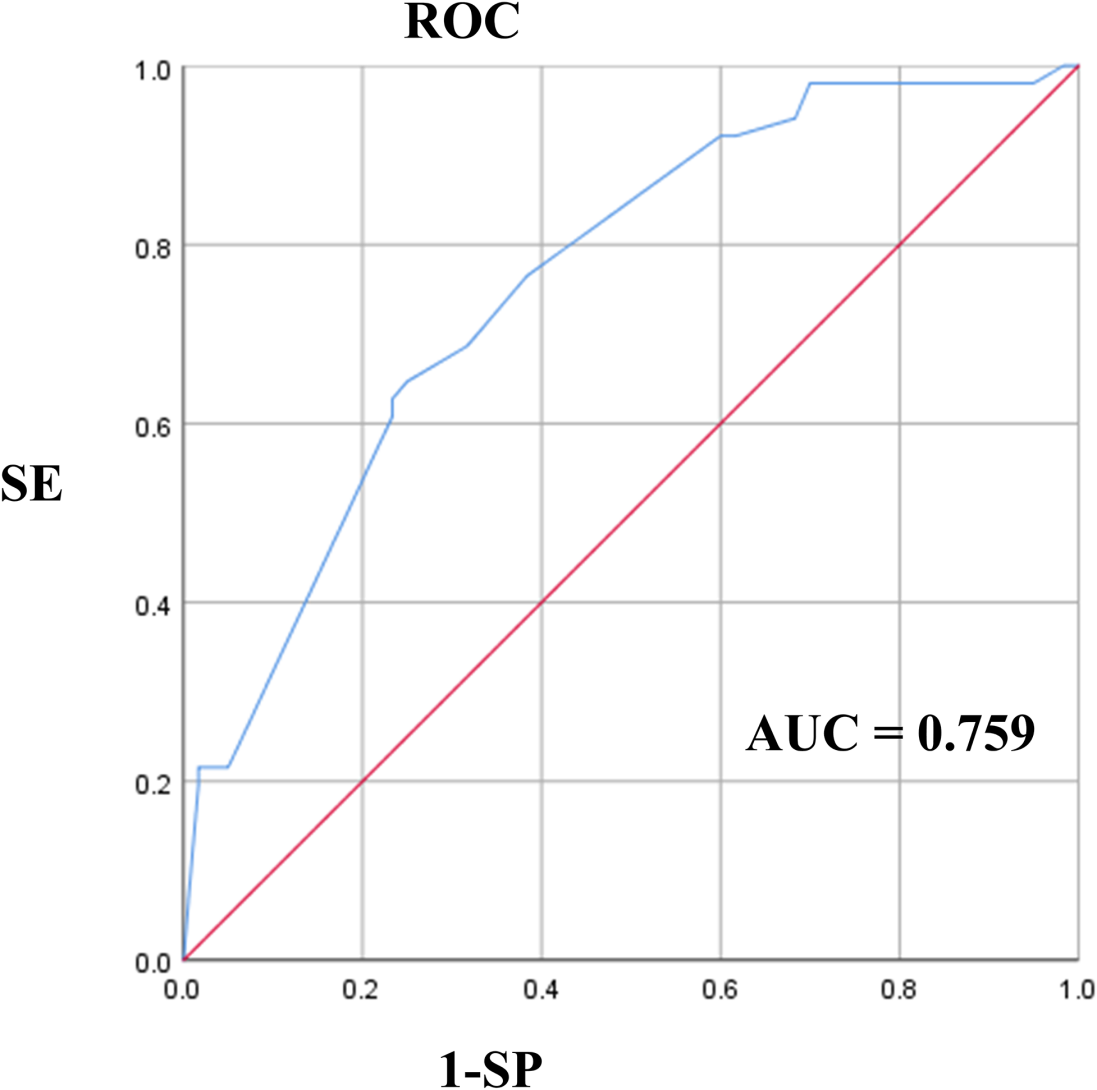
The ROC of multivariate logistic analysis in Model 1. ROC, Receiver Operating Characteristic Curve.

The results of the univariate analysis and multivariate analysis are shown in **Table 2**. Variables with a *p*-value less than 0.05 in univariate analysis included symptoms on admission (P=0.021), multifocality (P=0.020), composition (P=0.022), echotexture (P=0.037), and the presence of EF (P=0.020). After the above variables were included in the multivariate logistic regression, it was shown that symptoms on admission (P=0.045, OR=3.317), multifocality (P=0.012, OR=3.166) and solid lesions (P=0.012, OR=8.780) in MTC had a *p*-value less than 0.05. As shown in **Figure 4**, the AUC of multivariate logistic analysis is 0.759 in Model 1 (p<0.001).

**Table 2:** Univariate logistic regression and multivariate logistic regression of Group 1 and Group 2.

Variable	Univariate analysis		Multivariate analysis	
	*p-*value	OR (95% CI)	*p*-value	OR (95%CI)
Thyroid gland elasticity	0.246	1.875 (0.649-5.417)		
ETE	0.520	1.367 (0.528-3.538)		
Symptoms on admission	0.021	3.405 (1.199-9.672)	0.045	3.317 (1.029-10.693)
Duration of lesion detection	0.581	0.998 (0.990-1.005)		
Duration of symptoms	0.219	1.030 (0.982-4.523)		
Size of thyroid (maximum diameter)	0.399	0.861 (0.608-1.219)		
Size of lesions (maximum diameter)	0.973	1.005 (0.745-1.356)		
Multifocality	0.020	2.659 (1.168-6.065)	0.012	3.166 (1.284-7.806)
Orientation	0.299	1.522 (0.693-3.292)		.
Composition	0.022	6.125 (1.301-28.828)	0.012	8.780 (1.624-47.469)
Echotexture	0.037	2.965 (1.069-8.225)	0.083	2.782 (0.875-8.842)
Margin	0.512	0.727 (0.281-1.884)		
Echogenicity	0.137	1.882 (0.818-4.329)		
Vascularity	0.125	1.810 (0.849-3.859)		
EF	0.020	3.413 (1.202-8.219)	0.155	2.190 (0.743-6.452)
EFS	0.132	2.642 (0.745-9.368)		
Macrocalcifications	0.073	2.000 (0.937-4.268)		
FT3	0.154	0.648 (0.357-1.177)		
FT4	0.289	0.907 (0.757-1.087)		
TSH	0.156	0.836 (0.652-1.071)		
Anti-Tg	0.598	1.000 (0.998-1.001)		
Anti-TPO	0.353	0.999 (0.996-1.001)		
CEA	0.526	1.001 (0.998-1.004)		
Ctn	0.218	1.001 (1.000-1.002)		
CEA*	0.476	1.002 (0.996-1.008)		
Ctn*	0.063	1.001 (1.000-1.003)		

ETE, Extrathyroidal extension; EF, the presence of echogenic foci; EFS, the size of echogenic foci; FT3, Free Triiodo-thyroninen; FT4, Free Thyroxine; TSH, Thyroid-Stimulating Hormone; Anti-Tg, Anti-Thyroglobulin Antibody; Anti-TPO, Anti-Thyroid Peroxidase Antibody; CEA, Carcinoembryonic antigen; Ctn, Calcitonin; 95%CI, 95% Confidence Interval. *serological data after imputation.

The p - value of Little’s MCAR test is 0.477, indicating that the missing data of Ctn and CEA follow the Missing Completely at Random (MCAR) pattern in R. Taking into account relevant literature (18, 19) and the characteristics of our missing data, we selected the K - Nearest Neighbors (KNN) method for data imputation. The results reveal that the p - value of Ctn is 0.063 and that of CEA is 0.476 after imputation.

The p-value of the Hosmer-Lemeshow test in Model 1 was 0.336. The test passed (p-value > 0.05), indicating that the model was well calibrated.

**Figure 5:**
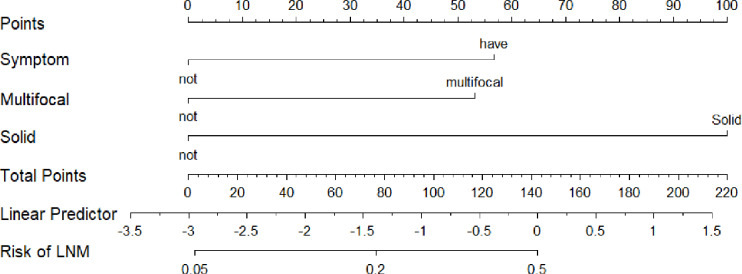
The nomogram of Model 1 to predict LNM. LNM, lymph node metastasis.

The nomogram of Model 1 is shown in **Figure 5**. The C-index of the nomogram to predict LNM was 0.707. The *p*-value of the nomogram is 0.0002 (p<0.001) and the R^2^ is 0.215.

### 3.4 Ultrasound diagnostic

#### 3.4.1 Ultrasound diagnosis of MTC

Among 111 lesions, 12 (12/111 = 10.81%) lesions were misdiagnosed as benign, and the remaining 99 (99/111 = 89.19%) were identified as abnormal lesions. 59 (59/111 = 53.15%) lesions clearly indicated malignancy, and the remaining 40 (40/111 = 36.04%) were identified as undetermined lesions but tending to be malignant among the 99 lesions.

#### 3.4.2 Ultrasound diagnosis of lymph nodes enlargement and LNM

**Table 3** presents the proportion of lymph node enlargement (LNE) identified by ultrasound in cohort 1.

**Table 3:** 2x2 contingency table for ultrasound diagnosis of LNE*.

Ultrasound diagnostic	Pathological results		Total
	Group 1	Group 2	
Positive 1	46	27	73
Negative 1	5	33	38
Total	51	60	111

*LNE, lymph node enlargement.

Based on the data in **Table 3**, each indicator was calculated and recorded in **Table 4**.

**Table 4:** Indicators for ultrasound diagnosis of LNE*.

	Sensitivity	Specificity	Accuracy	PPV	NPV	PLR	NLR
Value	90.20%	55%	71.17%	63.01%	86.84%	2.00	0.18

*LNE, lymph node enlargement; PPV, positive predictive value; NPV, negative predictive value; NLR, negative likelihood ratio; PLR, positive likelihood ratio.

Similarly, we recorded the data for positive 2 and negative 2 with a focus on clearly reported malignancy in **Tables 5** and **6**.

**Table 5:** 2x2 Contingency Table for Ultrasound Diagnosis of LNM*.

Ultrasound diagnostic	Pathological results		Total
	Group 1	Group 2	
Positive 2	26	2	73
Negative 2	25	58	38
Total	51	60	111

*LNM, Lymph node metastasis.

**Table 6:** Indicators for ultrasound diagnosis of LNM*.

	Sensitivity	Specificity	accuracy	PPV	NPV	PLR	NLR
Value	50.98%	96.67%	75.68%	92.86%	69.88%	15.29	0.51

*LNM, Lymph node metastasis. PPV, positive predictive value; NPV, negative predictive value; NLR, negative likelihood ratio; PLR, positive likelihood ratio.

### 3.5 Radiomics features and the comparison of three models

**Figure 6:**
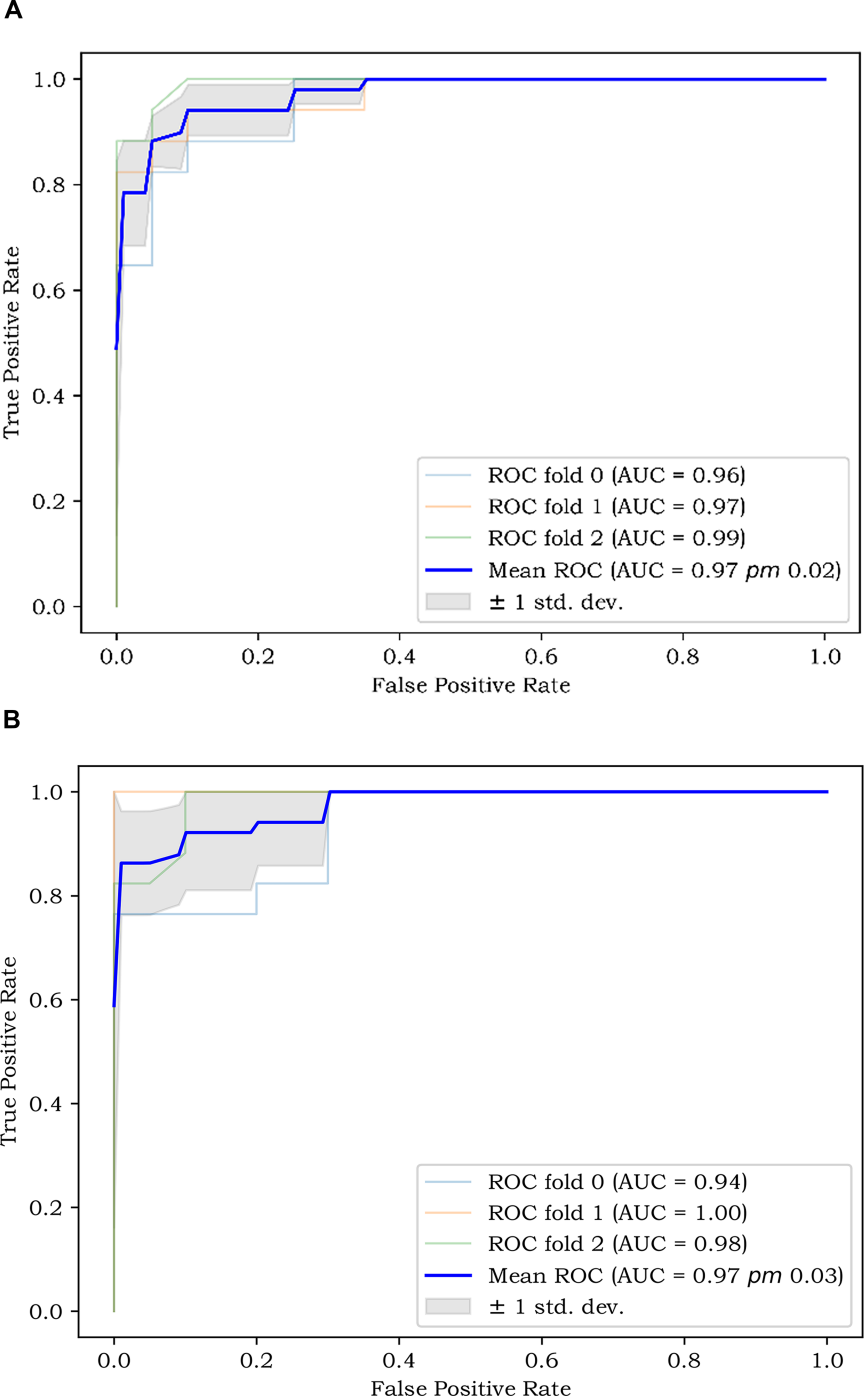
**(A)** The best ROC of Model 2; and **(B)** The best ROC of Model 3. ROC, Receiver Operating Characteristic Curve.

**Figure 7:**
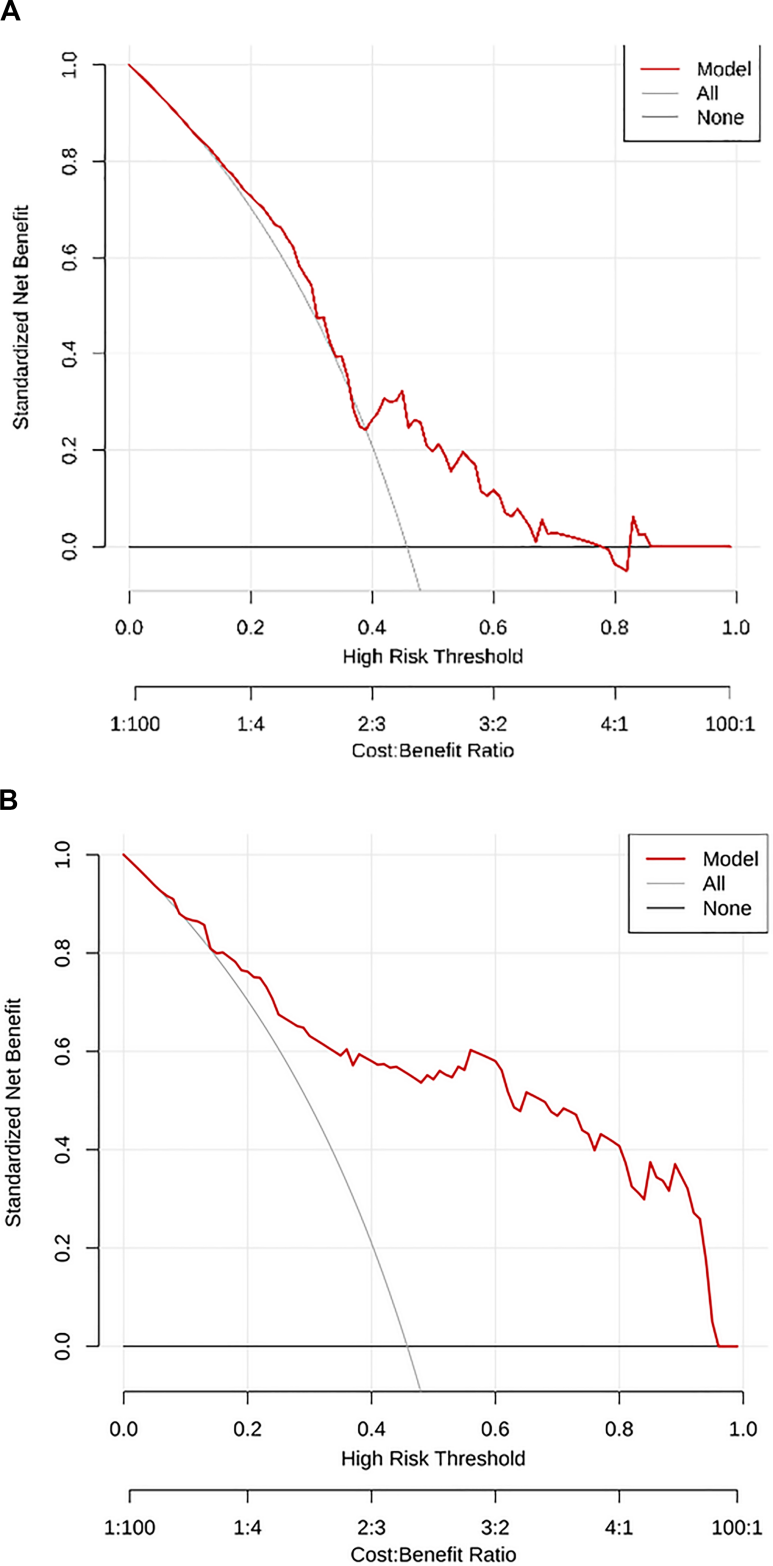
**(A)** The DCA curve of Model 2; and **(B)** The DCA curve of Model 3. DCA, Decision Curve Analysis.

A total of 1,125 features were extracted in radiomics analysis. Model 2 and Model 3 were implemented on the Darwin platform and yielded promising outcomes. The AUC values of Model 2 and Model 3 are presented in **Table 7**. As depicted in **Figures 6A, B**, the optimal ROCs of Model 2 and Model 3 are presented, respectively. Additionally, the DCA (Decision Curve Analysis) curves of them are shown in **Figures 7A, B**.

**Table 7:** The AUC values of Model 2 and Model 3*.

Model name	KNN	SVM	Logistic regression	Decision tree	GBT	Random forest	XGBOOST
Model 2	0.67	0.58	0.64	0.84	0.93	0.97	0.95
Model 3	0.80	0.85	0.85	0.87	0.92	0.97	0.94

*AUC, Area Under Curve; KNN, K - Nearest Neighbors; SVM, Support Vector Machine; GBT, Gradient Boosting Tree; XGBOOST, Extreme Gradient Boosting.

The p–value of Model 1 and Model 2 is 0.035. The p–value of Model 1 and Model 3 is 0.011. The p–value of Model 2 and Model 3 is 0.041.

## 4 Discussion

Our research found that patients with symptoms on admission (OR=3.317), multifocality (OR=3.166), and solid lesions (OR=8.780) in MTC may be at risk of LNM. Three predictive models were developed, with Model 3 emerging as the optimal one. Additionally, we explored the diagnostic performance of ultrasound for MTC lesions, as well as lymph node swelling and metastasis in MTC.

Symptoms on admission, one of the clinical factors, are related to LNM in our study. This finding is a major highlight of our research and rarely seen in previous studies. The reasons for the occurrence of symptoms are as follows. Partly, compressive symptoms occurred such as neck discomfort or pain due to the large size of the tumor or the malignancy progressing beyond the thyroid and infiltrating other cervical structures (20). And partly, fever and flushing occurred due to the neuroendocrine characteristics of MTC (21). The occurrence of symptoms may also be associated with distant metastasis, which may involve lymph nodes (22).

A meta-analysis (12)showed that multifocal lesions are one of the main risk factors for lateral cervical lymph node metastasis and central lymph node metastasis in MTC, which is consistent with our findings. The underlying cause may lie in the fact that multifocality might represent lymphatic infiltration in MTC (23). Therefore, if a patient has multifocal lesions, attention should be particularly paid to the presence of abnormal lymph nodes in ultrasound examinations.

Solid lesions in MTC are more likely to develop LNM compared to non-solid ones, with an OR of 8.780. They may carry not only a risk of malignancy according to ACR TI-RADS (24) but also a risk of LNM. Meanwhile, we have noticed that some lesions in MTC are non-solid (12.61%). These lesions include predominately solid, predominately cystic, cystic, and spongiform. Studies have shown (25) that cystic changes occur in MTC. So, attention should also be paid to the malignant identification of non-solid lesions.

The AUC of ROC is 0.759 and the C-index of the LNM nomogram in Model 1 is 0.707. These results indicate that a model with clinical, pathological, serological, and ultrasound features can predict LNM well. But the AUC of Model 3 is generally higher than that of Model 1 and Model 2. This shows that the extracted radiomics features are helpful for the diagnosis of LNM. When there are more features, the occurrence of LNM can be better predicted. We employed the k-fold cross-validation method, which more stably reflects model performance through multiple validations, reducing bias and overfitting.

There were only 12 lesions misdiagnosed as benign. Previous studies (25, 26) showed that the diagnostic rate of ultrasound for MTC is 72%-97%, consistent with our results. This suggests that ultrasound can identify abnormal thyroid lesions. But, only 59 (53.15%) lesions can be clearly indicated as malignant. The malignant category can be identified, but it is not specific enough to MTC. This may also mean that MTC has certain malignant features, but they are not obvious enough (27). Further research is needed on the differences between MTC and other malignant tumors of the thyroid.

Ultrasound rarely missed lymph node enlargement (5/111). It indicates that ultrasound can sensitively detect enlarged lymph nodes and there are few cases of occult LNM. Among 60 patients without LNM, only 2 cases were misdiagnosed as having LNM, demonstrating that ultrasound is unlikely to misdiagnose LNE as LNM. This indicates that the benign features of lymph nodes in MTC are evident. However, diagnosing LNM remains challenging due to insufficient specificity of LNM. The reason may be that the malignant features of LNM are not sufficiently distinct. Therefore, we need to pay attention to the combination of other examinations such as CT to enhance diagnostic accuracy.

However, it is interesting that the *p*-values of Ctn and CEA were greater than 0.05 in our study. Considering the clinical significance of CEA and Ctn (28), we performed imputation and conducted the univariate analysis. However, we still did not observe any statistically significant differences after imputation. We attribute these findings to the shortcomings inherent in a single-center retrospective study.

There are some limitations in our study as follows. First of all, a single-center retrospective study was conducted with a small sample size. It may lead to some bias in the results. Second, there was no distinction between central and lateral cervical lymph node metastasis due to the lack of available data. Finally, there was considerable missing data for CEA and calcitonin due to the inherent limitations of the retrospective study. The data after imputation can be used for statistical analysis, but it is not as good as the complete data.

We constructed three models using clinical variables combined with ultrasonographic features, incorporating the latest radiomics methods and also developed a nomogram with good predictive capabilities. Patients with symptoms on admission, multifocal characteristics and solid lesions in MTC might have an elevated likelihood of LNM. Symptoms could reflect real-world clinical urgency and tumor aggressiveness, fully highlighting the innovation and clinical significance of our research. Moreover, radiomics features were employed to further enhance the predictive ability. Additionally, we evaluated the diagnostic efficacy of ultrasound for MTC, lymph node enlargement and metastasis. Our models provide a robust foundation for predicting LNM in MTC, thereby aiding in personalized treatment decisions.

## Data Availability

The data analyzed in this study is subject to the following licenses/restrictions: The data that support the findings of this study are available from the corresponding author upon reasonable request. Requests to access these datasets should be directed to Huang Ying, huangying712@163.com.
